# Effects of Ferulic Acid Supplementation on Growth Performance, Carcass Traits and Histochemical Characteristics of Muscle Fibers in Finishing Pigs

**DOI:** 10.3390/ani11082455

**Published:** 2021-08-20

**Authors:** Nidia Valenzuela-Grijalva, Ismael Jiménez-Estrada, Silvia Mariscal-Tovar, Kenia López-García, Araceli Pinelli-Saavedra, Etna Aida Peña-Ramos, Adriana Muhlia-Almazán, Libertad Zamorano-García, Martín Valenzuela-Melendres, Humberto González-Ríos

**Affiliations:** 1 Centro de Investigación en Alimentación y Desarrollo, A.C (CIAD, A.C.), Hermosillo 83304, Sonora, Mexico; nvalenzuela@idiica.mx (N.V.-G.); pinelli@ciad.mx (A.P.-S.); aida@ciad.mx (E.A.P.-R.); amuhlia@ciad.mx (A.M.-A.); libertad@ciad.mx (L.Z.-G.); martin@ciad.mx (M.V.-M.); 2 Centro de Investigación y Estudios Avanzados del IPN, Departamento de Fisiología, Biofísica y Neurociencias, San Pedro Zacatenco, Mexico City 07000, Mexico; ijimenez@fisio.cinvestav.mx (I.J.-E.); smariscal@fisio.cinvestav.mx (S.M.-T.); 3 Departamento de Biología Celular y Fisiología, Unidad Periférica Tlaxcala, Instituto de Investigaciones Biomédicas, Universidad Nacional Autónoma de México, Chiautempan 90800, Tlaxcala, Mexico; kenia.lopezg@gmail.com

**Keywords:** phytogenics, growth promoters, pork production, ractopamine

## Abstract

**Simple Summary:**

Different compounds with potential phytogenic activity are being studied in the feeding of monogastrics and ruminants. The present study evaluated the effect of ferulic acid (FA) dietary supplementation on the growth performance, carcass quality and histochemical characteristics of the *Longissimus thoracis* muscle from finishing pigs. The growth performance, carcass quality, histomorphometric characteristics, and histochemical characteristics of skeletal muscle were affected by FA supplementation. The addition of FA on diet improved average daily gain, 12th rib fat depth and *Longissimus* muscle area in the same way as ractopamine treatment. Histochemical analysis indicated that FA treatment induces a shift in muscle fiber, a lower cross-sectional area, and a greater number of muscle fibers per area. The findings indicate that ferulic acid supplementation without extra-lysine can replace the use of ractopamine as a growth promoter in finishing pigs.

**Abstract:**

FA dietary supplementation on the growth performance, carcass traits and histochemical characteristics of the *Longissimus thoracis* muscle from finishing pigs was investigated. Four hundred and twenty pigs were used in this study, and 105 animals (with five replicate pens and 21 pigs per pen) were assigned to one of four treatments: basal diet (BD) without additives (C−); BD + 10 ppm ractopamine hydrochloride + 0.97% lysine (C+); BD + 25 ppm of FA (FA); and BD + 25 ppm of FA + 0.97% lysine (FA-Lys). Dietary supplementation with FA or ractopamine increased both the average daily gain (14%) and loin muscle area (19%), while fat deposition decreased by 53%, in comparison with C− (*p* < 0.05). The growth performance of pigs treated with FA was similar to those of ractopamine (*p* > 0.05). The histochemical analysis showed that FA and C+ treatments induced a shift in muscle fiber types: from fast fibers to intermediate (alkaline ATPase) and from oxidative to glycolytic fibers. Muscle tissues from animals treated with FA or ractopamine had a lower cross-sectional area and a greater number of muscle fibers per area (*p* < 0.05). Findings regarding growth performance and carcass traits indicate that FA supplementation at 25 ppm without extra-lysine can replace the use of ractopamine as a growth promoter in finishing pigs.

## 1. Introduction

Ractopamine hydrochloride is a β-adrenergic agonist (β-AA) analogue to natural hormones such as catecholamines, namely, norepinephrine or adrenaline, commonly used in the finishing diets of pigs with significant results in muscle development [1]. The mode of action of a β-AA is based on a recognition site into the β-receptors (β1 or β2) located in the cellular membrane of skeletal muscle fibers, where the agonist-receptor complex is generated, and the activation protein-kinase starts increasing the synthesis of proteins in skeletal muscle. β-AA compounds in animals cause a redirection of nutrients towards muscle mass, which improves the daily gain, feed conversion, and carcass yields [1]. At the level of muscle histochemistry, these compounds generate transitions from oxidative to glycolytic fibers and the higher size of muscle fibers caused by muscle hypertrophy [2].

The growth promotion response in finishing pigs by ractopamine supplementation is known to be conditioned by lysine level in the diet [3,4]. Several studies have recommended increasing the level of lysine to 0.97% to obtain better results on growth performance parameters [5,6].

On the other hand, due to the recently banned synthetic β-AA compounds and antibiotic growth promoters in several countries, the dietary supplementation of phytochemical compounds is a natural and sustainable potential strategy to improve animal growth [7,8]. The FA (4-hydroxy-3-methoxycinnamic acid) is a phytochemical present in a wide range of natural sources, such as grains and cereals. Due to its bioactive properties, FA has been utilized in its isolated form as an additive in foods and animal diets [9,10,11]. It has recently been shown to have a growth promoter effect in cattle and pigs and has been proposed as an alternative to β-AA used in these species [12,13,14,15]. Replacement of β-AA by phytochemical FA has its basis principally in two studies [16,17], which concluded that FA possibly interacts with β-adrenergic receptors (β-AR) producing a similar effect to β-AA and catecholamines. This interaction induces animal growth by a metabolism modification that increases protein deposition [18,19]. However, there are no studies that elucidate the mechanism of action of FA. Likewise, it is important to evaluate whether Lysine is a factor that limits animal growth when FA is used. In this way, we have hypothesized that FA can modify the histochemical and histomorphometric patterns due to β-AR stimuli on muscle fibers as occurs with ractopamine. Therefore, the aim of this study was to evaluate the effect of FA dietary supplementation (with two levels of lysine) or ractopamine on feedlot performance, carcass quality and histomorphometric parameters of fibers in the *Longissimus thoracis* of finishing pigs.

## 2. Materials and Methods

Experimental procedures involving finishing pigs were prepared according to the official standard guidelines and techniques for animal care approved in Mexico [20,21]. Additionally, the Research Ethics Committee of the Research Center for Food and Development (CIAD, México) approved and supervised all experimental procedures (CEI/003-2/2020).

### 2.1. Animals and Treatments

The experiment was conducted at a commercial pig production farm, located in the south of Sonora, Mexico (27°07′15″ N 109°44′40″ W). A growth performance trial was conducted with finishing pigs. Four hundred and twenty terminal animals (females and males), Landrace × Yorkshire crossbreed, were used (initial weight 67.6 ± 5 kg). Twenty pens with 21 animals per pen were completely randomized. Five pens were randomized for each treatment, as follows: negative control treatment (C−), basal diet (BD) without additives; positive control treatment (C+), BD + 10 ppm/kg of feed of ractopamine hydrochloride (Racmina^®^, PISA Agropecuaria, Guadalajara, Mexico) + 0.97% lysine; (FA) treatment, BD + 25 ppm/kg FA (Laboratorios Minkab, SA de CV, Guadalajara, Mexico); and (FA-Lys) treatment, BD + 25 ppm/kg FA + 0.97% lysine. All treatment groups were designed to be representative of a typical commercial finishing period. The feeding period was 27 days. Diets were based on sorghum and soybean meal and were formulated to meet all the nutrient requirements of finishing pigs according to NCR [22]. Experimental diets (Table 1) were formulated to be isocaloric (3.3 Mcal ME/kg). Lysine content in diets for C− and FA treatments was 6.5 g/kg, while for C+ and FA-Lys treatments it was 9.7 g/kg. Water and feed were ad libitum for all animals. The amount per ton of the FA or ractopamine was first incorporated into the mineral premix during diet preparation, and this premix was then incorporated to the rest of the experimental diet.

### 2.2. Growth Performance

All animals were individually weighed at the beginning (initial body weight, IBW) and the end (FBW) of the experimental period. The average daily gain (ADG) was estimated by the difference between initial and final weight divided by 27 (days of feeding period). The amount of feed offered and refused was weighed and recorded daily to estimate the feed intake (FI) by pen. Feed conversion per pen/treatment (FI/ADG) was also calculated. Animals were also observed daily throughout the study, and no adverse effects were detected related to C+, FA, and FA-Lys treatments.

### 2.3. Slaughter and Carcass Evaluation

At the end of the experiment, animals with a body weight above 113 kg (due to the fact that the producer receives from the buyer an economic prize for each kg of weight of those animals with a live weight above of this weight) were transported to the slaughterhouse (Frigorífico Kowi S.A de C.V., TIF 208, Navojoa, Sonora, México). Animals were slaughtered by electrical stunning according to Mexican Official Guidelines [21]. Carcasses were refrigerated for 24 h and subsequently 20 carcasses were randomized, selected and evaluated for the following traits (*n* = 20): back fat thickness (mm), loin muscle area (LM area, cm^2^) and marbling score at 12th intercostal space [23].

### 2.4. Sample Collection

After evisceration and before washing, within 30 min of slaughter at the abattoir, *Longissimus thoracis* muscle steaks (2.5 cm of thick) were collected from the right side of five carcasses per treatment (LT, at 8th thoracic vertebrae). Then, samples were sectioned transversely against muscle fiber into small pieces (1 × 0.5 cm) and each sample was placed on a cryo-mold. Samples for the histochemical analysis were submerged in 2-methylbutane (Sigma-Aldrich, St. Louis, MO, USA) and immediately frozen in liquid nitrogen and subsequently transported to the Departamento de Fisiología, Biofísica y Neurociencias at Centro de Investigación y de Estudios Avanzados del Instituto Politécnico Nacional (México city, México) and stored at −80° C until analysis.

### 2.5. Histomorphometric and Histochemical Analysis

Three serial transverse cryo-sections per muscle sample (10 μm thickness) were cut in a cryostat microtome (CM-1100; Leica Microsystems, Nussloch, Germany) at −20 °C, mounted on glass coverslips and stained. The oxidative metabolism of the muscle fibers was determined in consecutive muscle sections stained with the NADH-TR (nicotine-amide adenine dinucleotide tetrazolium reductase) histochemical technique modified from Nachlas et al. [24]. Muscle cryo-sections were incubated for 1 h at 37 °C in a NBT-NADH solution 1:1 (*v*/*v*) (1.2 mM nitro-blue tetrazolium (Sigma–Aldrich, St. Louis, MO, USA) diluted in 50 mM tris buffer (Biorad, Hercules, CA, USA), pH 7.6; 2.25 mM nicotinamide adenine dinucleotide (NADH; Sigma–Aldrich, USA) diluted in 50 mMtris buffer (BioRad, Hercules, CA, USA)). Subsequently, tissue sections were washed three times with deionized water. The excess of NBT-NADH solution was removed by washing with increasing and decreasing acetone concentrations (30, 60 and 90%). Samples then were washed three times for 5 min with deionized water and dehydrated. Cover slips were mounted with glycerol gel (2% gelatin, 50% glycerol, 0.5% phenol).

On the subsequent muscle slides, the myofibrillar actomyosin ATPase activity was observed using the alkaline ATPase technique (pH 9.4) with slight modifications [25]. Muscle sections were immersed in a pre-incubation solution (Tris base 10 mM (BioRad, USA) and 18 mM CaCl_2_ (J.T. Baker, México, México), pH 10.3) for 15 min. Afterward, samples were washed three times with deionized water and incubated for 1 h at 37 °C in 1.5% (*w*/*v*) adenosine 5-triphosphate (Sigma-Aldrich, USA), a pre-incubation solution, at pH 9.4. Muscle sections were washed with 200 mM CaCl_2_ for 3 min, transferred to 2% (*w*/*v*) CoCl_2_ (Sigma-Aldrich, Guillingham, UK) for 3 min, and then to 10% (*v*/*v*) ammonium sulfide (Sigma-Aldrich, USA) for 3 min. One more time, sections were washed, dehydrated and covered with a mounting medium (Cytoseal 60, Richard Allan Scientific, Kalamazoo, MI, USA) and coverslip.

Stained NADH-TR and ATPase muscle sections were examined using an optical microscope (Nikon Eclipse E600, Tokyo, Japan). Images were captured at 4 and 20 X magnifications using an Olympus C-5060 digital camera (Olympus Corp., Tokyo, Japan).

**Figure 1:**
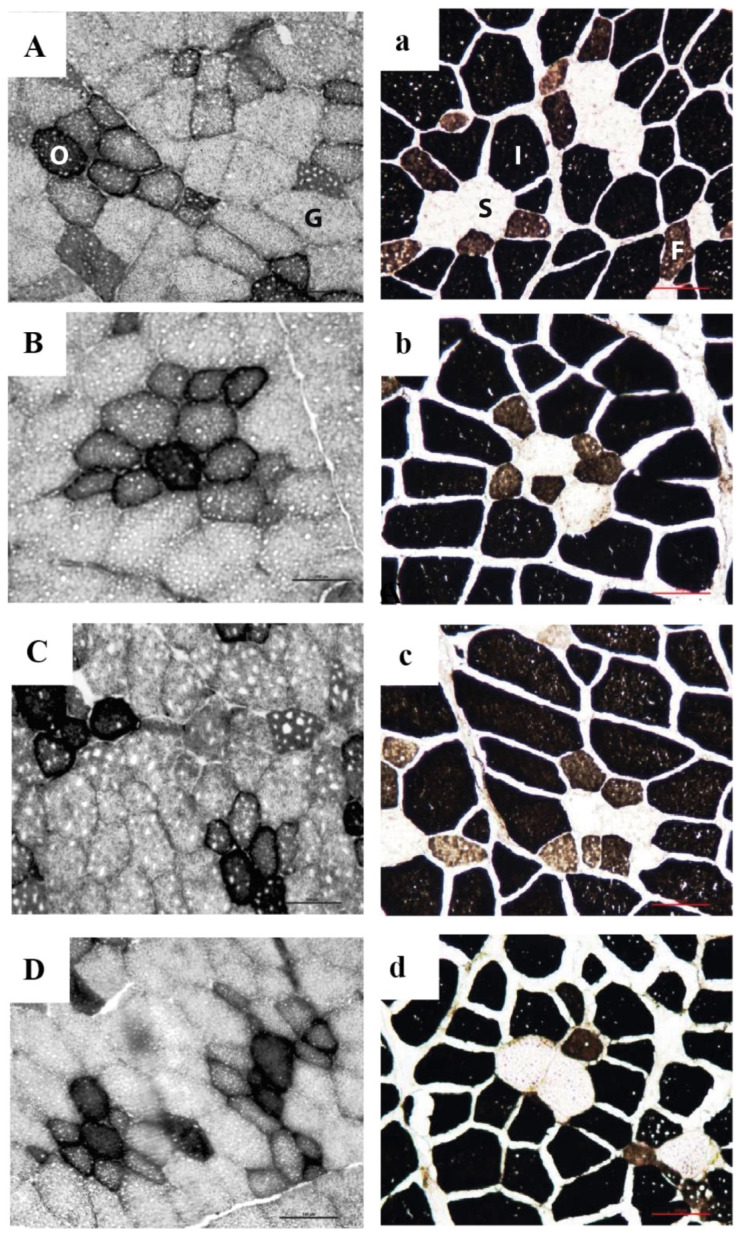
Transversal sections of Longissimus thoracis muscle per treatment. (**A**/**a**) negative control treatment (C−), without additive; (**B**/**b**) positive control treatment, 10 ppm/kg of feed ractopamine hydrochloride + 0.97% g lysine; (**C**/**c**) (FA) treatment, 25 ppm/kg of feed FA supplementation; and (**D**/**d**) (FA-Lys) treatment, 25 ppm/kg of feed FA supplementation + 0.97% lysine. NADH-TR technique (**A**–**D**), dark fibers were identified as oxidative (O) and light fibers as glycolytic (G); alkaline ATPase technique (**a**–**d**), light fibers were identified as slow (S, type I), dark fibers as intermediate (I, type IIb), and brown fibers as fast (F, type IIx/IId). Scale bar = 100 μm.

Four slides were prepared per muscle sample, and 3 images from each slide with the greatest muscle fiber integrity were obtained for histological parameters. Fiber type was classified according to the following criteria: for NADH-TR technique (Figure 1A–D), dark fibers were identified as oxidative (O) and light fibers as glycolytic (G); and with alkaline ATPase technique (Figure 1a–d), light fibers were identified as slow (S, type I), dark fibers as intermediate (I, type IIb), and brown fibers as fast (F, type IIx/ IId).

The total number of each fiber type was used to calculate the fiber type percentage per tissue section. Muscle fiber size per fiber type was evaluated by measuring the cross-sectional area (CSA); muscle fiber density was determined by counting the number of each fiber type in 40,000 μm^2^ of muscle sections. Subsequently, the total number of fibers in muscles was calculated by multiplying the fiber density from the loin-eye area (cm^2^) at the 12th rib. All histological parameters were measured using computer image analysis with the Image J 1.48v program.

### 2.6. Histological Data Analysis

The histogram comparison procedure [26] was used to generate histograms and to analyze the distribution of cross-sectional area for each fiber type within each experimental group.

### 2.7. Statistical Analysis

Animal randomization at each pen was performed by gender, weight, and genetic line using a complete randomized design. Growth performance data were analyzed using ANOVA-GLM, adjusting initial body weight as a covariate. The gender of the pigs was not significant in the ANOVA; therefore, it was eliminated from the statistical model. For carcass trait, ANOVA included final body weight as a covariate. Histological data were analyzed by one-way ANOVA for a completely random design. Means comparisons were carried out using orthogonal contrasts. The following specific contrasts were tested with the CONTRAST option of the software: contrast C1, C− vs. + FA + FA-Lys + C+; contrast C2, C+ vs. FA + FA-Lys; and contrast C3, FA vs. FA-Lys. Significances were estimated at a 0.05 probability level in error type I. All data were processed using the statistical package NCSS [26].

## 3. Results

### 3.1. Growth Performance and Carcass Traits

Dietary supplementation of FA (with and without lysine plus addition) or ractopamine in finishing pigs caused a significant effect (C1, *p* < 0.05) on productive parameters (Table 2). The ADG increased by approximately 13% in animals treated with FA or ractopamine as compared with the C− treatment (C1, *p* < 0.05). Animals supplemented with FA had similar ADG to C+ (C2, *p* > 0.05). Feed intake was not affected by FA or ractopamine supplementation, and the average for all treatments was 2.32 (*p* > 0.05).

Although supplementation with ractopamine or FA improved the feed conversion by 9%, in contrast to C- treatment (2.03 vs. 2.21), it was not significant (*p* > 0.05). No adverse effects were observed on the animals throughout the whole experimental period that could be associated with the compounds added to the diet (C+ or FA).

The LM area and back-fat depth were affected by treatments (*p* < 0.05). In both characteristics, the effect of the addition of FA or C+ was significant, increasing LM area by 19% and reducing the fat depth by 53% with respect to C- treatment (C1, *p* < 0.05). These characteristics were similar among FA and C+ (C2, *p* > 0.05).

### 3.2. Histochemical Analysis

The microphotography of transversal muscle sections stained with NADH-TR and alkaline ATPase techniques is illustrated in Figure 1. All treatments showed similar groups of fibers with the same metabolic characteristics. Generally, glycolytic fibers were surrounded by oxidative fibers.

### 3.3. Fiber Type Percentage

Fiber type compositions from alkaline ATPase and NADH-TR techniques were expressed as percentages (Table 3). Fiber type percentage (alkaline ATPase) was affected by treatments (*p* < 0.05). The percentage of fast fibers from animals with promoter (FA treatments and ractopamine) was approximately 77% lower than those muscle fibers from pigs without promoter (C1, *p* < 0.05). On the other hand, treatments with promoter had a higher percentage of intermediate fibers. For the same fiber type, similar percentages were found between the FA groups and ractopamine treatment (C2, *p* > 0.05). Percentage of intermediate fibers from muscle sections from FA-Lys treatment was significantly (C3, *p* < 0.05) higher (4%) than those from muscle sections from FA treatment.

Percentages of oxidative and glycolytic fibers (NADH-TR) were affected by growth promoter supplementation in the diet (C1, *p* < 0.05). Treatments with FA and ractopamine decreased the percentage of oxidative fibers and increased glycolytic fibers compared with C-. Nonetheless, a similar percentage of both fiber types (C2, *p* > 0.05) were observed between these treatments. Fiber type composition was not affected by lysine requirements in FA treatment (C3, *p* > 0.05).

### 3.4. Cross-Sectional Area (CSA)

CSA values from muscle sections stained with NADH-TR and alkaline ATPase techniques are shown in Table 3. Both growth promoter additive (contrast C1) and growth promoter additive type (contrast C2) had effects on all fiber types from alkaline ATPase staining (*p* < 0.05). Slow and intermediate fibers from animals treated with FA or ractopamine had lower CSA, while fast fibers from the same groups had a higher CSA. Slow and fast fibers from C+ had a significantly lower CSA than those from FA groups, whereas in the intermediate fibers there was a greater CSA in the C+, in comparison to the groups with FA (C2, *p* < 0.05). An effect of FA-Lys supplementation was found only on intermediate fibers (C3, *p* < 0.05), CSA being higher in fibers from FA-Lys in comparison with FA treatment. The CSA of oxidative fibers was not affected by treatments (*p* > 0.05). Glycolytic fibers (NADH-TR) from C- had the largest CSA value with respect to supplemented treatments (C1; *p* < 0.05).

### 3.5. Histograms

**Figure 2:**
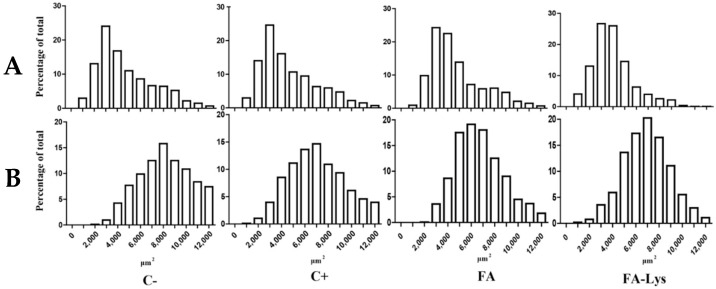
Cross-sectional area distribution patterns of muscle fibers (NADH-TR). (**A**) Oxidative fibers and (**B**) glycolytic fibers. Treatments: negative control treatment (C−), without additive; positive control treatment (C+), 10 ppm/kg of feed of ractopamine hydrochloride + 0.97% lysine; FA treatment, 25 ppm/kg of feed of FA; and FA-Lys treatment, 25 ppm/kg of feed of FA + 0.97% lysine.

Distribution frequency histograms of CSA were generated for oxidative and glycolytic fiber types (Figure 2). FA supplementation or ractopamine treatment caused a shift in the distribution of muscle fiber sizes. This effect was observed in the distribution frequency histograms, finding a higher quantity of smaller oxidative and glycolytic fibers in FA and FA-Lys groups, with respect to C− (<6000 μm^2^).

### 3.6. Muscle Fiber Density

The effects of FA supplementation on muscle fiber density and the total number of fibers are shown in Table 4. For fiber density, all contrasts were significant (*p* < 0.05). The lowest value was found in C−, in comparison to supplemented treatments (C1 effect, *p* < 0.05). Similarly, a higher density of fibers in C+ compared to treatments with FA was observed (C2 effect). FA-Lys supplementation resulted in a lower cellular density (6%) than that from FA treatment (C3, *p* < 0.05). The total number of muscle fibers was affected by the use of growth promoter (C1, *p* < 0.05) and the type of growth promoter (C2, *p* < 0.05). Muscle tissue from animals treated with FA or ractopamine had larger number of fibers than muscles from C− animals. However, muscle tissue from pigs with FA treatment had less fibers than animals with C+ treatment.

## 4. Discussion

### 4.1. Growth Performance and Carcass Traits

The replacement of growth promoters such as β-agonist and antibiotic growth promoters has been a great challenge for the meat industry for several years. Phytochemical compounds have been proposed as a natural alternative [27,28]. However, few phytochemicals have shown positive or at least similar effects to those triggered by traditional promoters on growth performance characteristics. Based on previous reports, it has been suggested that FA has the potential to be considered as growth promoter [14,16]. Therefore, in the current study, FA was tested as a replacement of ractopamine in finishing pigs. Both compounds, FA and ractopamine, showed an anabolic effect. It is well documented that β-AA ractopamine improves several productive and carcass parameters [29,30]. ADG and feed conversion are the most important production parameters. In this regard, those animals supplemented with FA (FA and FA-Lys) had similar ADG to those animals treated with ractopamine (a 19% increase respect negative control). This result suggests that FA could be used as a natural replacement of ractopamine in finishing pig production. A statistical meta-analysis performed by Apple et al. [29] showed that the use of ractopamine in finishing pigs at concentrations of 10 ppm increased (18%) the daily gain from 0.91 (control group) to 1.08 kg/d. Similar increases for both treatments (FA and ractopamine) were obtained in our study. In the case of FA supplementation, there are few reports that show the effect of FA supplementation on the growth performance of pigs and other productive animals. Some of these reports in ruminants are controversial. González-Ríos et al. [15] reported that the ADG of beef cattle supplemented with 6 ppm of FA increased in a similar proportion to animals supplemented with zilpaterol hydrochloride. On the other hand, Macías-Cruz et al. [31] found no effects on feedlot performance and blood metabolites in ewe lambs supplemented with 300 mg/d of FA during the last 30 days of feeding. These discrepancies in the response to FA supplementation by animal tissues may be associated to factors such as age, species, sex, genotypes, and others. In pigs, Herrera et al. [14] observed that productive parameters were not affected by 15 ppm FA supplementation; however, such results may be associated with the low concentration of FA used in that study. In another study, FA supplementation of heifers increased average daily gain (ADG) by 21%, hot carcass weight by 1.8% and cold carcass weight by 1.6% with respect to the control [32].

Growth promotion response in finishing pigs by ractopamine supplementation is known to be conditioned by lysine level in the diet [2,4]. Several studies have recommended increasing the level of lysine to 0.97% to obtain better results on growth performance parameters [5,6]. In this respect, we decide to evaluate the effect of this recommended concentration in FA-Lys treatment, and to observe if this lysine level was necessary to promote growth. This study demonstrated that 0.97% lysine supplementation was not necessary for FA supplementation to produce similar effects on ADG as ractopamine supplementation. This effect may be related to the mechanism of action of FA to promote animal growth. However, to our knowledge, the action mechanism of FA in pigs has not been elucidated.

The success of traditional growth promoter supplementation by the meat industry is based on the capacity to improve several productive parameters not only at the farm level but also at all levels of the meat chain. Specifically, the effects on carcass traits have been well documented [19,33]. As shown by several studies, ractopamine inclusion in the diet of finishing pigs increased the LM area by about 25% and decreased the fat deposition by 19% [5,29]. In the current study, it was observed that FA supplementation had a similar response to ractopamine. Animals supplemented with FA increased LM area by 19%, while subcutaneous fat deposition was decreased by 53%. Moreover, in a similar manner to the ADG parameter, the effect of FA on carcass traits was not dependent on lysine supplementation. Conversely, Li et al. [10] did not find any effect of FA supplementation (100 ppm) on subcutaneous fat deposition in pigs. Similarly, Dávila-Ramírez et al. [34] did not find any effect of the addition of a mixture of plant extracts on dorsal fat of growing-finishing pigs.

In general terms, total intramuscular fat content, marbling, and back fat thickness are parameters negatively affected by the use β-AA compounds [18]. The above shows the lipolytic mechanism of this type of compound. In this study, we propose that ferulic acid presents a similar mechanism to β-AA, this being based on the chemical structure, effect on increased protein deposition, increased rib eye area and changes in muscle histology. So, under this assumption, ferulic acid would negatively affect intramuscular fat deposition similar to β-AA. In the same way, the use of this phytochemical, much like ractopamine, decreased the thickness of the back fat compared to the control group. (C1 significant, 7.02 vs. 14.11 mm).

Despite the fact that β-AA compounds improve several carcass traits, marbling is still a parameter negatively affected by growth promoters [3]. For some meat consumers, one of the most important factors affecting the purchasing decision is the amount of intramuscular fat [35]. In our study, FA supplementation decreased subcutaneous fat deposition without affecting marbling scores, obtaining a better score than others reported by ractopamine [29,36]. Similarly, the meta-analysis carried out by Andretta et al. [5] indicated that ractopamine significantly reduces backfat thickness, but without affecting intramuscular fat content.

### 4.2. Histochemical Characteristics

Muscle fiber type composition is responsible for a variety of properties of skeletal muscle and meat, including contractile and sensory characteristics, respectively [37]. In pig muscles, there are four muscle fiber types designated by their predominant myosin heavy chain isoform: I (slow), IIA, 2X (D), and IIB [38].

All skeletal muscles have adaptive potential, which means that they are capable of altering their structure (mainly its fiber composition) in response to diverse stimuli, such as hormonal profile and exercise [39]. The transition pathway between different fiber types is well documented: slow (I) ↔ IIA ↔ IIX ↔ IIB [39]. There are few studies focused on evaluating the effect of ractopamine over the histological characteristics of porcine muscles [40,41]. In the case of “repartitioning agent” supplementation, it increases muscle growth by modifying their muscle fiber type composition [40,41]. Based on previous studies, we hypothesized that dietary inclusion of FA in the feed of finishing pigs will act as a “repartitioning agent” as ractopamine does. In this trial, ractopamine and FA treatments altered fiber type composition (both staining methods) by increasing the intermediates type percentage and reducing the contribution of other fiber types.

For β-AA compounds, Li et al. [40] and Paulk et al. [41] observed that ractopamine treatment (7.5 and 10 ppm, respectively) increased the percentage of type IIX fibers in the pig *Lonsgissimus dorsi* muscle. Likewise, they found an increment on cross-sectional area that can be responsible for muscle growth. The increment in faster fiber types and reduced protein turnover are the main mechanisms by which β-adrenergic agonists induce muscle growth [42]. When a compound causes a shift in muscle fiber type composition in animals for meat production, it is considered as a modulator of animal growth [43]. Parr et al. [44] mentioned that slow aerobic fibers appear to be resistant to anabolic stimuli, while fast glycolytic fibers seem to be more receptive to growth stimuli than the other types of muscle fibers (slows and fast oxidative fibers). In this sense, several studies suggested that β-AAs specifically target fast fibers due to the increase in the CSA in this fiber type [45]. Contrastingly, in the current study, the CSA value of all muscle fiber types of supplemented (FA and ractopamine) animals was smaller than control animals, which suggests that not only the fast fiber type seems to be receptive to growth stimuli.

For other phytochemicals, Mizunoya et al. [46] found that a small dose (0.5% *w*/*w*) of dietary apple polyphenols upregulates slow MyHC isoforms in lower hind-limb muscles of 12-week-old male Sprague-Dawley rats. The same polyphenols at a higher dose (5% w/w) shifted from fast to intermediate/slow in the MyHC isoforms [47]. Similarly, in this trial, a shift in muscle fiber type was observed, finding an increase in the percentage of intermediate fiber type. Peña et al. found that FA supplementation of lambs increases fiber size, without affecting the meat quality parameters [33].

In pigs, classic histochemical staining techniques identified IIB fiber types and IIX (D) as one fiber type [48]. Therefore, the results reported here must be interpreted with caution because the contribution of these fiber types is approximately 80% of the total [49]. Future research would need immunohistochemistry, electrophoresis or “in situ” hybridization analysis to properly characterize the MyHC isoforms present in muscle fibers of the pig. It is important to note that supplementation with ferulic acid at the dose used in this study was safe and practical for use as a phytochemical growth promoter on finishing pigs.

The ATPase and NADH-TR techniques have shown a transition from the slow to fast fiber type. According to its metabolic characteristics, slow fibers have high lipid content, while fast fibers have a low content [50]. Relating to these characteristics observed through histological techniques with carcass traits such as marbling, a decrease in intramuscular fat deposition is expected in animals supplemented with a growth promoter (Ractopamine or FA). However, in this study, marbling was not negatively affected by the use of these additives. Nevertheless, the decrease in the backfat thickness in supplemented animals is consistent with the histological findings, an increase in intermediate (ATPase) and glycolytic (NADH-TR) fibers. These findings strengthen the hypothesis of the possible ferulic acid mechanism of action such as that in β-AA compounds. On the other hand, from the point of view of the meat industry, it is important, since not affecting the marbling could maintain the sensory characteristics of the meat, such as tenderness.

Skeletal muscle enlargement in adult animals has been attributed to a fiber hypertrophy phenomenon, which primarily increases the fiber cross-sectional area parameter [51,52]. Some studies related to the use of growth promoters in animal production have reported that the fiber CSA of animals with a growth promoter treatment was larger than those of animals without promoters, suggesting a fiber hypertrophy pathway increasing muscle mass [53,54]. However, evidence obtained in other studies indicates that fiber hyperplasia contributes to the increase in muscle mass in adult animals as a part of their development, although this is controversial [55]. Since in our study muscle fiber CSA significantly decreased with supplementation (Table 3), our results coincide with the later proposal because we observed a significant increase in the number of muscle fibers in the pig LT muscle in response to FA and ractopamine. In addition, Kelley [51] suggested that in diverse animal species, certain stimuli, such as a mechanical overload, may increase the fiber type number in muscles. Administration of β-AA and FA induced an increment in muscle growth; however, contrary to previous reports [52], we found that a hyperplasic phenomenon is acting on the muscle. Both treatments (FA and ractopamine) promoted the incidence of fibers with smaller CSA (Figure 2). This effect suggests that those treatments induced a proliferative state, a hyperplasia effect, on muscle tissue in which the muscle fibers likely did not have sufficient time to reach a similar size to muscle fibers from control animals at the moment of slaughter. The results obtained for histograms clearly agreed with this proposal, since smaller CSA values were observed in FA treatments.

Lysine is one of the most important amino acids involved in the control of protein metabolism, participating actively in the generation of body proteins, peptides, and non-peptide molecules [4]. It has been recognized that the addition of lysine during ractopamine supplementation considerably increases the production performance of pigs [3]. However, our findings indicate that extra lysine supplementation is not a condition for muscle mass gain for ferulic acid supplementation. It is very important to point out this finding, because it is a great alternative to reduce feeding costs.

## 5. Conclusions

Dietary FA supplementation in finishing pigs can contribute to improving growth performance and carcass parameters without the need to increase the lysine level in diet (0.97%) as when using ractopamine as a growth promoter. Therefore, FA supplementation at 25 ppm can be an adequate replacement of ractopamine as a growth promoter. Since the growth performance and carcass characteristics clearly agreed with the histological evaluations made in this study, the observed changes in muscle fiber composition, CSA and the muscle fiber density allow for novel cellular mechanisms by which FA induces muscle growth. However, the mechanism underlying FA activity requires clarification. Further investigations are necessary to delineate how individual muscle fibers and β-adrenergic receptors participate in modulating such a phenomenon.

## Figures and Tables

**Table 1:** Ingredient composition and calculated nutrient composition (g/kg as-fed) of experimental diets.

	Treatment ^a^			
Item	C−	C+	FA	FA-Lys
Sorghum	899	796	899	796
Soybean meal (47% PC)	75	177	75	177
Soybean oil	5	7	5	7
Calcium carbonate	7.5	6	7.5	6
Dicalcium phosphate	5.3	5.7	5.3	5.7
Salt	3.5	3.5	3.5	3.5
Threonine	1.2	1.1	1.2	1.1
Methionine	0.3	0.5	0.3	0.5
Vitamin premix ^b^	2.4	2.4	2.4	2.4
Mineral premix ^c^	0.8	0.8	0.8	0.8
Ferulic acid, ppm	-	-	25	25
Ractopamine hydrochloride, ppm	-	10	-	-
**Calculated composition**				
Dry matter	893.8	894.2	893.8	894.2
ME (MCal/Kg)	3.341	3.341	3.341	3.341
Protein	139.5	168	139.5	168
Total Lysine	6.5	9.7	6.5	9.7
Calcium	6.2	6.6	6.2	6.6
Phosphorus	5.7	6.1	5.7	6.1

^a^ Treatments: negative control treatment (C−); positive control (C+), ractopamine hydrochloride 10 ppm/kg of feed + 0.97% lysine; FA, treatment with 25 ppm/kg of feed of FA; and FA-Lys, FA 25 ppm/kg of feed + 0.97% lysine. ^b^ Vitamin premix; each kg diet provided: 80 mg DL-tocoferol acetate; 2200 mg retinol acetate; 16.5 mg colecalciferol; 4.4 mg menadione sodium bisulphite; 242 mg choline; 33 mg niacin; 8.8 mg riboflavin; 24.2 mg D-pantothenic acid; and 0.04 mg vitamin B12. ^c^ Mineral premix; each kg fed provided: 70 mg Fe; 70 mg Zn; 27 mg Mn; 8 mg Cu; 0.38 mg I; 0.2 mg Se.

**Table 2:** Growth performance and carcass traits of finishing pigs supplemented with FA or ractopamine.

Item ^a^	Treatment ^c^					Significance		
						Contrast ^e^		
	C−	C+	FA	FA-Lys	SEM ^d^	C1	C2	C3
IBW, kg	64.39	67.94	70.11	68.01	2.96	0.596	0.472	0.348
FBW, kg	96.06	99.86	99.62	100.22	0.99	0.004	0.967	0.672
ADG, kg	1.05	1.19	1.18	1.20	0.03	0.004	0.960	0.677
Feed intake, kg DM	2.31	2.45	2.40	2.12	0.06	0.145	0.395	0.427
Feed conversion, kg DM	2.21	2.09	2.02	1.98	0.07	0.071	0.396	0.431
LM area, cm^2^	44.96	54.64	52.45	54.90	1.55	<0.001	0.605	0.286
12th-rib fat depth, mm	14.11	8.0	6.44	6.62	0.90	<0.001	0.184	0.891
Marbling score ^b^	3.11	2.66	3.0	2.87	0.25	0.374	0.392	0.736

^a^ IBW, initial body weight; FBW, final body weight; ADG, average daily gain; and LM area, loin muscle area. ^b^ Marbling score using NPPC (1999). ^c^ Treatments: negative control treatment (C−), without additive; positive control (C+), ractopamine hydrochloride 10 ppm/kg of feed + 0.97% lysine; FA, treatment with 25 ppm/kg of feed of FA; and FA-Lys, FA 25 ppm/kg of feed + 0.97% g lysine. ^d^ Pooled SEM (standard error of mean), *n* = 5 pens/treatment. ^e^ Contrast: C1, C− vs. FA + FA-Lys + C+; C2, C+ vs. FA+ FA-Lys; C3, FA vs. FA-Lys. For carcass traits *n* = 20/treatment.

**Table 3:** Percentage of fibers and cross-sectional area (CSA, μm^2^) for each skeletal muscle fiber types in finishing pigs supplemented with FA or ractopamine.

Item	Treatment ^a^					Significance		
						Contrast ^c^		
	C−	C+	FA	FA-Lys	SEM ^b^	C1	C2	C3
Percentage								
*ATPase*								
Slow	11.38	9.82	10.47	8.33	0.85	0.056	0.988	0.113
Intermediate	73.24	86.56	86.11	89.58	0.75	<0.001	0.286	0.004
Fast	14.92	3.84	3.95	2.31	0.86	<0.001	0.359	0.189
*NADH-TR*								
Oxidative	22.11	13.28	12.54	11.63	0.88	<0.001	0.110	0.209
Glycolytic	77.89	86.71	87.45	88.37	0.87	<0.001	0.117	0.214
CSA								
*ATPase*								
Slow	9256.37	6245.05	7425.88	7101.65	187.40	<0.001	<0.001	0.347
Intermediate	4818.71	4494.67	3595.15	4042.73	128.89	<0.001	<0.001	0.025
Fast	3409.51	3291.65	3735.61	3909.43	102.69	0.013	<0.001	0.284
*NADH-TR*								
Oxidative	8490.29	7907.06	6816.84	7000.20	578.19	0.071	0.120	0.567
Glycolytic	5050.46	3800.18	3720.56	3888.66	250.63	<0.001	0.895	0.811

^a^ Treatments: negative control treatment (C−); positive control (C+), ractopamine hydrochloride 10 ppm/kg of feed + 0.97% lysine; FA, treatment with 25 ppm/kg of feed of FA; and FA-Lys, FA 25 ppm/kg of feed + 0.97% lysine. ^b^ Pooled SEM (standard error of mean) of simple-effect means *n* = 5 by treatment. ^c^ Contrast: C1, C− vs. FA + FA-Lys + C+; C2, C+ vs. FA+ FA-Lys; C3, FA vs. FA-Lys.

**Table 4:** Muscle fiber density and total number of fibers from LM area of finishing pigs with FA or ractopamine (alkaline ATPase staining).

Item	Treatment ^a^					Significance		
						Contrast ^c^		
	C−	C+	FA	FA-Lys	SEM ^b^	C1	C2	C3
Density (40,000 μm^2^)	51.92	72.42	70.78	66.30	1.13	<0.001	0.015	0.016
Number of fibers ^d^	5,913,047	10,016,050.21	9,237,457	8,916,402	134,083.26	<0.001	<0.001	0.368

^a^ Treatments: negative control treatment (C−), basal diet; positive control (C+), ractopamine hydrochloride 10 ppm/kg of feed + 0.97% lysine; FA, treatment with 25 ppm/kg of feed of FA; and FA-Lys, FA 25 ppm/kg of feed + 0.97% lysine. ^b^ Pooled SEM (standard error of mean) of simple-effect means *n* = 5 by treatment. ^c^ Contrast: C1, C− vs. FA + FA-Lys + C+; C2, C+ vs. FA+ FA-Lys; C3, FA vs. FA-Lys. ^d^ Total number of fibers in LM area.

## Data Availability

Tha data presented in this study are available on request from the corresponding author (H.G.-R).
